# Anti-Tumor Effects of Ginsenoside 20(S)-Protopanaxadiol and 1,25-Dihydroxyvitamin D3 Combination in Castration Resistant Prostate Cancer

**DOI:** 10.3390/medicines8060028

**Published:** 2021-06-04

**Authors:** Mohamed Ben-Eltriki, Subrata Deb, Gehana Shankar, Gray Meckling, Mohamed Hassona, Takeshi Yamazaki, Ladan Fazli, Mei Yieng Chin, Emma S. Tomlinson Guns

**Affiliations:** 1 Vancouver Prostate Centre at Vancouver General Hospital, 2660 Oak Street, Vancouver, BC V6H 3Z6, Canada; gehanashankar@gmail.com (G.S.); treiki_1041@yahoo.com (G.M.); mohdhessein@yahoo.com (M.H.); takeshi.yamazaki@gmx.com (T.Y.); lfazli@prostatecentre.com (L.F.); chinmeiyieng@yahoo.ca (M.Y.C.); emmabluetube@gmail.com (E.S.T.G.); 2 Therapeutic Initiative, Department of Anesthesiology, Pharmacology and Therapeutics, Faculty of Medicine, University of British Columbia, Vancouver, BC V6T 1Z3, Canada; 3 Department of Pharmaceutical Sciences, College of Pharmacy, Larkin University, 18301 N. Miami Avenue, Miami, FL 33169, USA

**Keywords:** 1,25-dihydroxyvitamin D3, 20(S)-protopanaxadiol, ginsenoside, prostate cancer, vitamin D receptor, xenograft, combination, antitumor

## Abstract

In spite of possessing desirable anticancer properties, currently, limited clinical success has been achieved with 20(S)-protopanaxadiol (aPPD) and 1,25-dihydroxyvitamin D3 (calcitriol). This study is designed to evaluate if the combination of aPPD with calcitriol can inhibit human prostate cancer xenograft growth by using nuclear receptor signaling. Athymic male nude mice were utilized to establish an androgen-independent human prostate cancer C4-2 cell castration-resistant prostate cancer (CRPC) xenograft model. Mice were treated orally for six weeks with 70 mg/kg aPPD administered once daily or three times per week with 4 µg/kg calcitriol or in combination or only with vehicle control. Contrary to our expectations, calcitriol treatment alone increased C4-2 tumor growth. However, the addition of calcitriol substantially increased aPPD-mediated tumor growth suppression (76% vs. 53%, combination vs. aPPD alone). The combination treatment significantly increased levels of cleaved caspase-3 apoptotic marker compared to vehicle-treated or aPPD-treated C4-2 tumors. The mechanistic elucidations indicate that tumor inhibition by the aPPD and calcitriol combination was accompanied by elevated vitamin D receptor (VDR) protein expression. In silico data suggest that aPPD weakly binds to the native LBD pocket of VDR. Interestingly, the combination of aPPD and calcitriol activated VDR at a significantly higher level than calcitriol alone and this indicates that aPPD may be an allosteric activator of VDR. Overall, aPPD and calcitriol combination significantly inhibited tumor growth in vivo with no acute or chronic toxic effects in the C4-2 xenograft CRPC nude mice. The involvement of VDR and downstream apoptotic pathways are potential mechanistic routes of antitumor effects of this combination.

## 1. Introduction

Prostate cancer (PCa) is the most common male cancer type identified globally [1]. Though PCa is curable if it is diagnosed and treated early, a significant number of patients progress to a more advanced stage termed as castration-resistant prostate cancer (CRPC) [2,3,4,5,6,7,8]. The currently available treatment options for CRPC are limited and still represent a therapeutic challenge. Since androgens are one of the main driving forces of prostate cancer, other than chemotherapeutic agents such as docetaxel and cabazitaxel, the androgen receptor (AR) and steroid biosynthesizing cytochrome P450 17A1 (CYP17A1) have been the primary targets of PCa treatment [9]. However, hormonal agents or chemotherapeutic agents have their unique challenges in negotiating the CRPC molecular pathways or the intense nonspecific cytotoxicity. In addition, the overall survival period following treatment of CRPC is often limited to less than six months [10,11]. Identification of novel agents that work through non-AR or non-hormonal pathways are needed to provide a multifaceted treatment approach. The compounds derived from natural sources are relatively safer and, due to their pleiotropic nature [12,13], they have the capability to efficaciously inhibit prostate cancer development and progression.

**Figure 1:**
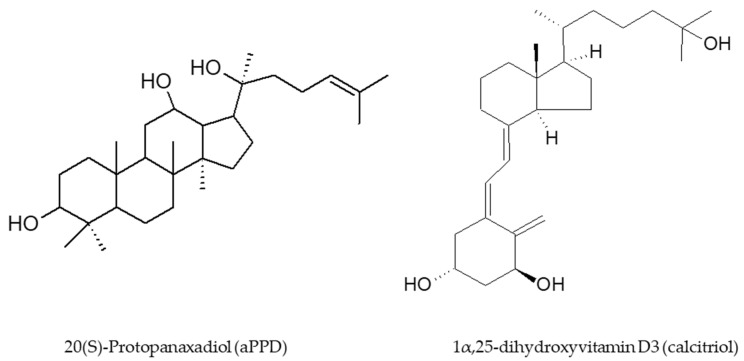
Chemical structures of 20(S)-Protopanaxadiol (aPPD) and 1α,25-dihydroxyvitamin D3 (calcitriol).

Ginseng-based products have been very commonly used as complementary and alternative medicine by individuals worldwide [14]. Ginsenoside triterpenoid saponins are responsible for the pharmacological activities of ginseng [15,16]. Depending on the non-sugar aglycone portion of the structures, ginsenosides can be categorized as the 20(S)-protopanaxadiol (aPPD) type (e.g., Rh2, Rc, Rbl, and Rb2) (Figure 1) and 20(S)-protopanaxatriol (aPPT) type (e.g., Rh1, Rgl, Re, and Rf) [17]. The deglycosylation of ginsenosides that generates individual aglycones by intestinal bacteria in acidic gastric pH is a key step [18,19]. Due to this bioconversion, aglycones (e.g., aPPD) are more bioavailable than the parent ginsenosides (e.g., Rh2) [20]. Among several ginseng derivatives, aPPD has been characterized as one of the most potent ginsenoside metabolite with reported anticancer effects in prostate cancer, breast cancer, and leukemia [21,22,23,24]. The work from our laboratory has demonstrated that aPPD can demonstrate anticancer effects in androgen-dependent LNCaP and androgen-independent C4-2 cells in vitro and inhibits PCa xenograft growth in preclinical models [21,22,25,26,27,28,29,30]. In addition, aPPD has the antioxidant and anti-inflammatory properties, which can positively affect the anticancer mechanism [31]. The optimum hydrophilic and lipophilic properties of aPPD facilitate its ability to cross biological membranes, interacts with nuclear receptors, and the stimulation of signaling pathway [32]. The aPPD achieved a peak plasma concentration of 4.9 µM with a half-life of 28 min in a PC-3 mouse xenograft model of PCa [22,30].

1,25-dihydroxyvitamin D3, which is typically known as calcitriol, is the most active form of vitamin D3 with the ability to cross cellular membrane [33]. The formation of calcitriol from the prohormone vitamin D3 possesses at least two enzymatic reactions catalyzed by cytochrome P450 (CYP) enzymes [34]. In addition to the regulation of serum calcium levels, previously published preclinical and clinical reports have demonstrated that calcitriol is the most promising natural vitamin D3 derivative in inhibiting the development and progression of several cancer types [26,35,36]. Indeed, the work from our laboratory has shown anticancer activities of calcitriol in androgen-dependent LNCaP and androgen-independent C4-2 prostate cancer [21]. The underlying mechanisms of calcitriol-mediated anticancer effects encompass pro-apoptotic, pro-differentiating, anti-proliferative, and anti-inflammatory actions [35,37]. Vitamin D receptor (VDR) is naturally the primary regulator of calcitriol effects with its expression and activity facilitating anticancer effects in PCa [26,36,38,39]. Calcitriol is known to function as paracrine and autocrine agents and the ubiquitous expression of VDR in tissues further encourages that mechanism [37]. In spite of the promising biological characteristics, until now calcitriol has limited success primarily due to its hypercalcemic effects at higher concentrations [40].

The prevention and treatment of cancer with a single agent has been replaced with the use of combination regimens [41]. Due to the heterogeneity of PCa pathogenesis and ability of CRPC tumors to survive in challenging conditions, the combination of agents is a more promising approach to thwart cancer development and progression. The combination of anticancer agents can target independent molecular pathways or can also interact at the pharmacokinetic or pharmacodynamic level. In the context of the present study, although aPPD has a wide tissue and tumor distribution, it experiences limited bioavailability [22]. On the other hand, calcitriol doses need to be curtailed to minimize the adverse effects of hypercalcaemia. Thus, in spite of desirable anticancer properties as an individual agent, until now aPPD and calcitriol have limited clinical success. From previous in vitro work, it can be discerned that these agents have the potential to interact at the VDR and CYP protein levels [21,42]. Thus, the purpose of the present study was to determine the antitumor effects of aPPD and calcitriol in combination with androgen-independent C4-2 xenograft CRPC mouse model in vivo. The effects of the addition of calcitriol to aPPD in CRPC tumor suppression and cooperative VDR action were studied using VDR protein expression, activity, and in silico docking analyses.

## 2. Materials and Methods

### 2.1. Test Compounds and Reagents

Ginsenoside aPPD (MW 460.73 g/mol) was provided as a gift by the Shanghai Innovative Research Center of Traditional Chinese Medicine (Shanghai, China). Its purity was confirmed in our laboratory to be ~98.9% as determined by LC-MS/MS. Calcitriol solution (1 µg/mL) was purchased from the Vancouver General Hospital Pharmacy (Vancouver, BC, Canada). Human VDR Kit was purchased from INDIGO Biosciences Inc. (State College, PA, USA). All other chemicals were obtained from Sigma-Aldrich (Oakville, ON, Canada).

### 2.2. Xenograft Preparation and Animal Treatment with Oral Gavage

All animal experiments were conducted as per the University of British Columbia’s Committee on Animal Care protocols and protocol # A11-0377 held by Dr. Guns at the Vancouver Prostate Centre (2014). Male athymic mice (Harlan Sprague Dawley, Inc., Indianapolis, IN, USA) of age 6–8 weeks old and weighing 25–31 g were utilized in our experiments. C4-2 cells (BD Biosciences, Oakville, ON, Canada) were then subcutaneously inoculated at the posterior dorsal site as described previously [43,44]. Mice were castrated and treatments were initiated when serum PSA values reached more than 25 ng/mL. Eight mice per group were used once the total tumor size exceeded 100 mm^3^. The aPPD was administered once orally on a daily basis at a dose of 70 mg/kg (117–150 µL) alone or in a combination with calcitriol 3 times per week dosed at 4 µg/kg (100–128 µL) or calcitriol alone or the vehicle control at an equivalent volume based on weight. Dose selection was based on previous work completed in our lab for safety and was formulated just prior to oral administration [22]. Briefly, the aPPD was solubilized in ethanol: propylene glycol: water (2:7:1, *v/v/v* ratio) was given by oral gavage at a dose of 70 mg/kg (highest achievable dose, limited due to gavage volume limitations (150 µL) implemented by the institutional animal care committee). The calcitriol solution solubilized in ethanol: propylene glycol: water (2:7:1, *v/v/v* ratio) was administered orally alone and in combination with the aPPD (aPPD 70 mg/kg) and calcitriol 4 µg/kg (100–128 µL).

### 2.3. Tumor Growth and Toxicity Assessment

Tumor volume measurements were taken twice every week. Calipers were used to measure the three perpendicular axes of each tumor (volume = ¼ length × width × weight × 0.5326) [25]. During the treatment period, animals were monitored twice per week for changes in body weight (g) and monitored daily for the appearance and signs of acute toxicity including death, lethargy, blindness, and disorientation. Mice had to be sacrificed when body weight loss exceed >20% or tumor volume exceeded 1500 mm^3^. After 46 days of treatment approximately 18 h after their last treatment dose, all mice were euthanized using CO_2_ along with cervical dislocation. Blood samples were collected (obtained by cardiac puncture) for CBC and the chemistry profile (liver enzymes and kidney function tests, serum electrolytes, glucose, serum albumin, and total blood protein levels). In addition, the liver, spleen, kidney, and lung tissues were collected for further toxicological and histopathological analysis.

### 2.4. Tumor Collection

Mice were sacrificed at the end of the study (46 days) with tumors harvested in two portions: either preserved in 10% formalin buffer and tissue sections embedded in paraffin blocks for histopathological analysis or frozen in liquid nitrogen and stored at −80 °C for protein analysis. Preparation of paraffin-embedded tissue sections and immunohistochemical analyses were carried out as previously described [45,46].

### 2.5. In Silico Docking between aPPD and VDR

AutoDock 4 (Scripps Research, La Jolla, CA, USA) [47] was employed for the in silico docking. The X-ray crystal structure of VDR LBD complexed with calcitriol was obtained from the Protein Data Bank (PDB ID: 1IE9). The protein model was prepared with Molecular Operating Environment (MOE) 2015.1001 [C3] by adding the missing residues and the side chains to the protein coordinate in the X-ray structure [48]. The center of the binding pocket was defined based on the coordinate of calcitriol ligand in the X-ray structure and the box dimension of 24 Å × 24 Å × 24 Å was used for the grid search, which is large enough to accommodate the ligand molecule. In order to explore the potential alternative binding places of aPPD on the VDR LBD surface, a box dimension of 96 Å × 96 Å × 96 Å was prepared for the grid search, which is large enough to accommodate the entire VDR LBD surface and in silico docking simulation was performed.

### 2.6. Western Blotting for VDR

Excised C4-2 tumor tissue was homogenized as per the manufacturer’s protocol, using the Precellys™ tissue homogenizer system (Bertin Technologies, Montigny le Bretonneux, France). Proteins were extracted using a RIPA buffer as previously described [21]. Briefly, tumor tissue (100 mg) was homogenized in RIPA buffer with 1× protease inhibitor at a 1:4 (tissue: buffer) ratio using Precellys™ Tissue Homogenizing CKMix (Cat. # 3961-1-009) at 6000 rpm for two cycles of 20 s each with a 15 s break. Protein assay and quantification were performed as described previously [21]. Thirty to forty micrograms of protein were loaded per lane into 12% SDS-acrylamide gels. Mouse monoclonal antibody for beta actin was used as loading control (1:5000; Sigma-Aldrich, Oakville, ON, Canada) and rabbit polyclonal antibodies for VDR (1:200; Santa Cruz Biotechnology Inc., Dallas, TX, USA) were used to develop the immunoblots. Conjugated secondary antibodies (anti-mouse IRDye 800 at a dilution of 1:5000 and anti-rabbit IRDye 680 at a dilution of 1:20,000) were used and obtained from Cedarlane Laboratories (Burlington, ON, Canada).

### 2.7. VDR Transactivation Assay

The assays were performed according to the manufacturer’s suggested protocol (Indigo Biosciences Inc., State College, PA, USA). The effect of aPPD in concentrations ranging from 0.032 to 500 µM alone or in combination with calcitriol on VDR activity was analyzed after 24 h. Briefly, the reporter cells were first prepared in Cell Recover Media as a suspension and then 100 mL of this suspension was cultured in 96-well assay plates and incubated at 37 °C for 24 h. We performed both agonist and antagonist assays and calcitriol (reference compound-VDR agonist) was provided in the kit and used as a positive control. The aPPD were diluted using compound screening medium and added into the reporter cells. Cells were treated with either 0.1% DMSO (solvent control) or serial dilutions of increasing concentrations of aPPD alone and in combination with 0.5 nM calcitriol. At 24 h after treatment, the medium was aspirated and 100 µL/well of Luciferase Detection Reagent was added. The chemical oxidation of luciferin into oxyluciferin by the luciferase is accompanied by light production that was quantified as luminescence by a TECAN M200Pro instrument to determine VDR activity. Each concentration was assayed in duplicate, with a biological replicate of *n* = 3. The toxicity of treatments on VDR reporter cells was assessed in the same experimental conditions using the 3-(4,5-dimethylthiazol-2-yl)-5-(3-carboxymethoxyphenyl)-2-(4-sulphophenyl)-2H-tetrazolium (MTS) assay [21].

### 2.8. Assessment of Apoptosis by Immunohistochemistry Analysis

All tissue preparation of paraffin-embedded sections and immunohistochemical analyses of cleaved-caspase 3 C4-2 tumors were performed as previously described [45,46]. Specifically, C4-2 tumors were sectioned and stained with hematoxylin and eosin (HE) and the desired areas marked along with their corresponding paraffin blocks. The antibody for cleaved-caspase 3 (Asp175) (5A1E) (#9664, 1:50, rabbit anti-human) was procured from Cell Signaling Technology (Danvers, MA, USA). All sections used for immunohistochemistry were lightly counterstained with 5% (*w/v*) Harris hematoxylin. Five fields of each slide were randomly chosen and their were images taken (400) using an AxioCam HR CCD mounted on an Axioplan 2 microscope and Axiovision 3.1 software (Carl Zeiss, Toronto, ON, Canada). Positively stained cells and whole cells in each image were counted and the percentage of positive cells was calculated. The TMAs were manually constructed (Beecher Instruments, Silver Spring, MD, USA) by punching quadruplicate cores of 1 mm for each sample giving a total of 144 cores. All scoring was performed blind with respect to treatment by LF and based on relative immunoreactive intensity on a four-point scale.

### 2.9. Statistical Analysis

A one-way ANOVA test followed by a Tukey Test was used to determine if there was a difference between the mean values of treatment groups. The level of significance was set prior at a *p* value of <0.05.

## 3. Results

### 3.1. Calcitriol Sensitizes Castration-Resistant C4-2 Tumors to aPPD Anticancer effects

**Figure 2:**
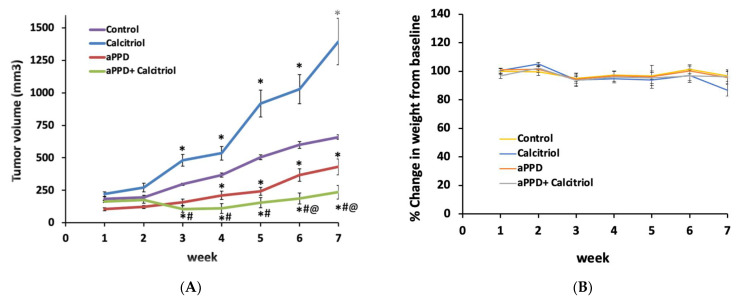
The antitumor effects of aPPD either alone or in combination with calcitriol on the tumor volume of C4-2 mice xenograft (**A**) and body weight (**B**). Change in tumor volume was followed over time for mice treated orally with either the control (ethanol: propylene glycol: water at 2:7:1) or aPPD (70 mg/kg once daily) or a combination of aPPD and calcitriol (4 µg/kg three times weekly) formulation. In vivo toxicity as assessed by the decrease in mean body weight of C4-2 mice xenograft. No animals showed any signs of toxicity or weight loss. Data are presented as Mean ± SEM (*n* = 8). A *p* value < 0.05 was considered significant when compared to vehicle control (*) or calcitriol-treated group (#) or aPPD-treated group (@).

The anticancer effects of aPPD and calcitriol combination were assessed in nude mice bearing human C4-2 prostate tumors. Contrary to our expectations, calcitriol treatment alone increased tumor growth in C4-2 tumors in this study (Figure 2A). However, the mice treated with the combination showed significant inhibition of tumor growth from week three onwards. The combination therapy induced additional blockade of tumor development relative to the mice treated with aPPD itself or with vehicle (Figure 2A). At the end of the treatment period, the addition of calcitriol to aPPD led to significantly higher blockade of tumor development compared to aPPD alone (76% by combination vs. 53% by aPPD, *p* < 0.01).

### 3.2. Lack of Toxicity from aPPD and Combination Treatment

No significant difference was observed in the mice body weight among the treatment groups (Figure 2B). Histopathological examinations of the liver, spleen, lung, and kidney showed no signs of abnormal findings between the groups (data not shown). There was no statistical difference in serum albumin, alkaline phosphatase, and alanine aminotransferase values. However, in the mice treated with the combination there was an increase in serum calcium levels compared to the controls (Supplementary Table S1). Serum creatinine levels were determined as a measure of kidney toxicity and the results suggest that there was no significant difference in serum creatinine levels between the groups (Supplementary Table S1). Overall, there were no significant differences in the organ-specific biochemical pathological tests and histopathological findings between the groups in any of the tissues examined. Therefore, the treatments were safe at the doses used in this study.

### 3.3. Induction of VDR Protein Expression by aPPD and Combination

The VDR protein expression was determined using Western blot analyses following treatment with aPPD and calcitriol or aPPD alone. VDR protein expression was strongly upregulated in the C4-2 xenograft tumors from aPPD-treated or combination-treated mice.

**Figure 3:**
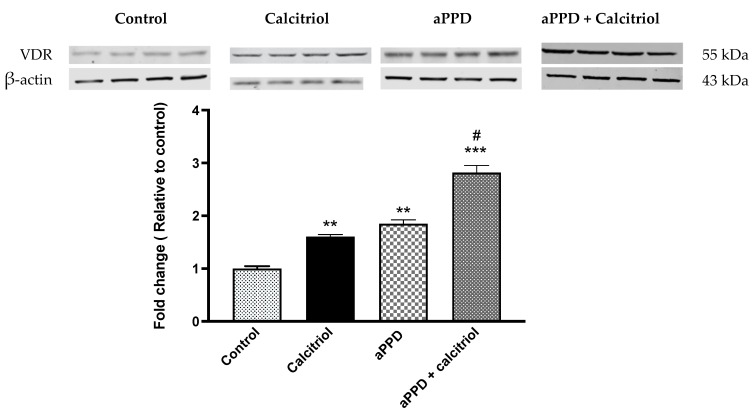
Vitamin D receptor (VDR) protein levels as studied by Western blot analysis of C4-2 xenograft tumor. The Western blot experiments were independently performed twice and a representative immunoblot involving four mice per treatment group is shown here. The fold change data are presented as Mean ± SEM of four mice (*n* = 4) in each treatment group. A *p* value < 0.05 was considered significant (#), a *p* value < 0.01 was considered extremely significant (**) and a *p* value < 0.001 was considered extremely significant (***) change compared to vehicle control (*) or calcitriol or aPPD-treated group (#).

The relative quantification of VDR protein to beta actin shows that calcitriol or aPPD increased VDR protein levels by approximately 2-fold in C4-2 tumors (*p* < 0.001) than when compared to the control group, while substantial additional increases in VDR expression were observed when calcitriol was added to aPPD (*p* < 0.05). (Figure 3).

### 3.4. In Silico aPPD Binds to VDR

**Figure 4:**
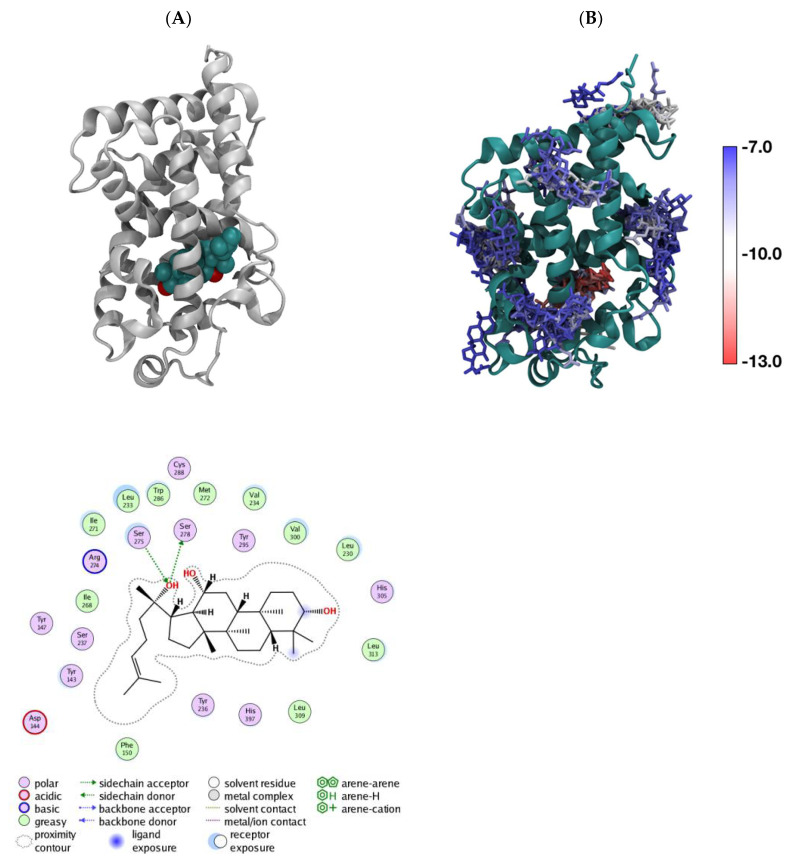
Predicted docking poses of aPPD (green) in VDR in the 1IE9 X-ray structure. LBD (**A**), the superimposed binding poses of aPPD around VDR LBD with binding strengths between −12.77 kcal/mol (in the native LBD pocket) and −7.5 kcal/mol (**B**); each aPPD is colored based on its binding strength.

In order to explore the potential binding site of aPPD on VDR, we performed in silico docking simulation by targeting the LBD binding site. Figure 4 presents the predicted docking poses of aPPD (green) in the VDR LBD in the 1IE9 X-ray structure. In silico docking predicted that the binding strength of calcitriol is −13.51 kcal/mol while that of aPPD is shown here to be 12.77 kcal/mol (Figure 4); this suggests that aPPD is a slightly weaker binder to the native LBD pocket compared to calcitriol.

### 3.5. Combination Enhances VDR Transactivation

**Figure 5:**
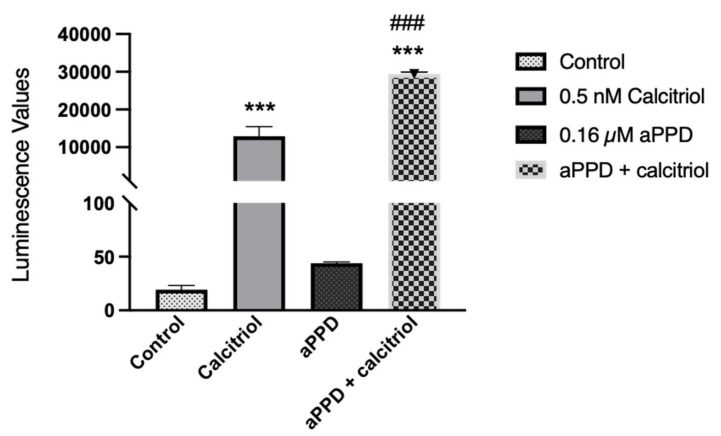
VDR transactivation following calcitriol (0.5 nM), aPPD (0.16 µM), or in combination in the reporter cells. Data are expressed as luminescence values following treatment and normalized to the blank assay kit as instructed by the manufacturer. The results represent the mean ± SEM of three independent experiments. A *p* value < 0.001 was considered an extremely significant (*** or ###) change compared to the media control (*) or calcitriol or aPPD-only treated group (#).

The VDR transactivation assay was carried out with the reporter cells to assess the ability of aPPD, either alone or in combination, to activate the receptor. We examined the effect of aPPD on VDR activation and cellular toxicity either alone or in combination with calcitriol. Following treatment of reporter cells with 0.032 µM to 500 µM of aPPD or calcitriol alone, aPPD showed limited to no activation of VDR but calcitriol demonstrated a strong concentration-dependent activation starting with as low as 0.032 nM (data not shown). Interestingly, in the presence of aPPD (0.16 µM), calcitriol-mediated (0.5 nM) VDR activation was enhanced by approximately 2-fold compared to calcitriol-alone (Figure 5). The higher aPPD concentrations (0.8 µM or 4 µM) did not increase calcitriol-mediated VDR activation any further than with 0.16 µM of aPPD (data not shown). The MTS cell viability assay indicates that combinations of calcitriol (0.5 nM) and aPPD up to 4 µM did not exhibit any cellular toxicity determined after 24 h incubation. However, combinations with aPPD at ≥20 µM demonstrated significantly decreased cell viability (data not shown).

### 3.6. Induction of Apoptosis by the Combination

**Figure 6:**
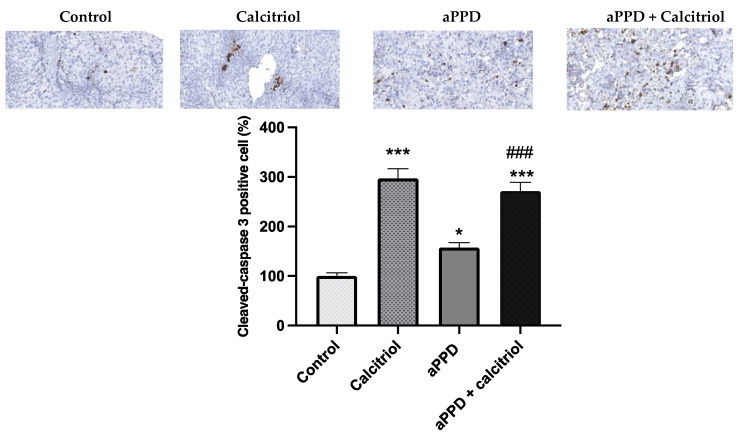
Immunohistochemistry staining of tumors derived from C4-2 xenografts. Apoptosis marker cleaved caspase-3 in the tumors. C4-2 cell xenograft tumors were excised after 46 days of treatments with aPPD and aPPD and calcitriol combination or control. Data are presented as Mean ± SEM (*n* = 4). A *p* value < 0.05 was considered significant (*) and a *p* value < 0.001 was considered an extremely significant (*** or ###) change compared to vehicle control (*) or to aPPD treated group (#).

The C4-2 tumors were harvested from mice following 46 days of treatment (once daily) and were subjected to immunohistochemical analyses for cleaved caspase-3. The immunohistochemistry results suggest that the treatment with calcitriol-alone or with the combination of aPPD and calcitriol both markedly increased cleaved-caspase 3 positive cells (*p* < 0.001) (Figure 6). Treatment with aPPD alone modestly increased caspase-3 levels compared to the control group but the number of caspase-3 positive cells was much lower than the calcitriol-alone or combination groups.

## 4. Discussion

Based on our work with LNCaP and C4-2 prostate cancer cells and the observation that calcitriol can sensitize the effects of aPPD [21], the xenograft study was designed. The combination of calcitriol and aPPD was evaluated for antitumor effects in an androgen-independent C4-2 xenograft CRPC mouse model in vivo. In the present study, the ability of the combination to lower tumor volume and to interact with VDR and its downstream pathways has been assessed.

Our results exhibit that aPPD can inhibit the PCa xenograft tumor in mice and additions of calcitriol further lowered the tumor volumes. The suppressive effect of aPPD was observed as early as after seven days of treatment; however, the addition of calcitriol to aPPD demonstrated its enhancement effects starting on day 35. The addition of calcitriol to aPPD treatment regimen resulted in the substantially greater inhibition of tumor growth by the combination than aPPD treatment alone (76% vs. 53%, respectively, on day 46). It is noteworthy that either the aPPD or combination group did not display any acute or chronic toxicity in the xenograft mice as assessed from the mean body weight, physical appearance, and behavior of dietary intake. Similarly, biochemical tests for organ-specific functions carried out at the end of the treatment period indicate safer profiles for aPPD and calcitriol. Previously, we have shown that aPPD and its ternary solvent system (ethanol: propylene glycol: water; 2:7:1, *v/v/v* ratio) that was used to formulate the agents in the recent study is non-toxic at lower volumes [22,29,30]. The safety data are consistent with the previous in vivo work with aPPD and calcitriol in mouse models [27,28,49]. This is the first report of calcitriol and aPPD combination antitumor effect in an androgen-independent C4-2 xenograft CRPC mouse model in vivo. The absence of any body weight loss indicates that, rather than non-specific cytotoxicity, the effects of the combination are more molecular in nature.

It is intriguing to note that the calcitriol-alone group appears to experience increased tumor volume. The lack of growth inhibition by calcitriol in C4-2 xenograft tumors may indicate complex tumor microenvironment, loss of balance between intrinsic pro-cancerous vs. anticancerous pathways, and severity of castration resistance in this model. It is important to recognize that there are previous reports that indicate that vitamin D and its derivatives can demonstrate dual action depending on the length of treatment, model, and other pathophysiological factors. For example, Ajibade et al. (2014) reported that early intervention with calcitriol in TRAMP mice model led to reduced tumor proliferation. However, prolonged calcitriol treatment resulted in the development of a resistant and significantly more aggressive disease associated with increased distant organ metastasis, which is in agreement with our observation in the current study [50]. Ajibade et al. (2014) also reported that castration resistant PCa was unresponsive to vitamin D intervention, which may be due to aggressive nature of the castration resistant phenotype [50]. In addition, calcitriol enhanced the metastatic potential of 4T1 mouse mammary gland cancer [51]. Similarly, calcitriol failed to exert antiproliferative effects on the vitamin D insensitive cells derived from transgenic adenocarcinoma of mouse prostate [52]. In summary, depending on the experimental model, complexity of the tumor microenvironment, and treatment period calcitriol by itself can potentially fail to lower mouse tumor xenograft volume.

VDR is central to the molecular mechanism of calcitriol and could be the driving force for the combination effects seen in the current study. For calcitriol, VDR is the natural and most potent target but the potential of aPPD to modulate VDR is the crucial question here. The aPPD significantly increased VDR protein levels by two-fold compared to the control, while the combination increased protein expression by three-fold in C4-2 tumors. It is interesting to note that a modest increase in VDR expression by calcitriol-alone was unable to cause any tumor inhibition effect. However, significantly higher VDR expression as a potential additive effect of the calcitriol and aPPD combination led to tumor growth suppression. The VDR protein expression data are consistent with our previously published in vitro work with C4-2 human prostate cancer cells [21]. It is possible that a threshold level of VDR protein expression is required before VDR-mediated antitumor effects can be observed. Previous studies with human tissues highlighted that the higher expression of VDR in tumors was associated with less aggressive cancer and a low risk of cancer related deaths. Individuals that carried tumors with the highest VDR expression had significantly reduced risk of developing lethal prostate cancer or prostate cancer [53,54]. It is worth highlighting here that AR is a negative regulator of VDR expression in CRPC cells. Mooso et al. (2010) have shown that the increase in AR expression caused a decrease in VDR levels, which was mediated through the shared coregulators [55]. Calcitriol can increase AR protein expression in LNCaP prostate cancer cells [55], while we have previously shown that aPPD significantly inhibited AR protein expression and activities in vivo in the C4-2 xenograft mouse model [25]. Cao et al. (2014) reported suppression of full length and splice variant AR expression in castration-resistant 22Rv1 xenograft tumors [28]. Taken together, in the current study it is plausible that the sensitivity of C4-2 tumors to the anticancer activities of the combination was enhanced when a balance of VDR and AR expression was achieved through the actions of aPPD.

Subsequently, the novel finding of aPPD-mediated increase in VDR expression was followed up by VDR transactivation assay to determine if aPPD can activate VDR. Interestingly, our results suggest that aPPD very weakly activates VDR in reporter cells at concentrations up to 500 µM; however, the addition of calcitriol stimulates VDR activity by approximately 650-fold with more than a two-fold difference between calcitriol-only and combination groups. Thus, it can be ascertained that the stimulation of VDR activation by aPPD and calcitriol cannot be ascribed only to calcitriol. The additional two-fold (~16,000 unit) increase following addition of aPPD to calcitriol suggests that aPPD is potentially activating VDR through non-ligand binding site(s) in a cooperative manner with calcitriol. It is also plausible that aPPD increases VDR protein expression and thus offers a higher amount of protein for calcitriol binding and stimulated downstream signaling. Chemically, aPPD has a steroidal nucleus with a side chain, while calcitriol is a secosteroid with a disjointed B-ring and a side chain [34,56] (Figure 1). In spite of the structural differences in the ring structure, it is possible that aPPD interacts with VDR albeit at a site that is not responsible for the direct activation of the receptor. To our knowledge, this is the first report of VDR activation by aPPD and calcitriol as a combination. Although, either in vitro or in vivo, there are not many examples of non-vitamin D ligands of VDR; Khedkar et al. [57] reported non-secosteroidal VDR agonists without hypercalcemic effects.

The in silico aPPD docking of aPPD to VDR LBD was carried out to determine if aPPD was able to bind to VDR. The aPPD prefers to weakly bind to the native LBD pocket in addition to other potential binding places. It was observed that aPPD binds to the area around Helix 2 that is associated with the A-Pocket which is a proposed alternative pocket [58,59]. Compared to calcitriol, aPPD is a weaker binder to the native LBD pocket. However, irrespective of the binding ability of aPPD to VDR, low or limited activation of VDR by aPPD by itself suggests lack of functional effect of aPPD and VDR LBD binding. Nonetheless, the higher VDR activation by aPPD and calcitriol compared to calcitriol-alone indicates that aPPD potentially binds to VDR pockets responsible for receptor cooperability. These results suggest that aPPD could be an allosteric activator that binds to VDR at multiple non-active sites which leads to a change in VDR conformation and increase in affinity to calcitriol. Therefore, we postulate that there may be an effect of aPPD in enhancing calcitriol-mediated VDR activation and ultimately VDR-mediated tumor growth suppression. Alternatively, VDR has the ability to work through cooperative mechanisms as reported by previous studies [60]. Based on the available information, it is reasonable to investigate whether aPPD can bind to other pockets on VDR protein such as pocket A as previously proposed [58,59,61] and whether it can activate VDR signaling. To our knowledge, this is the first report of in silico aPPD docking to the different domains of VDR.

**Figure 7:**
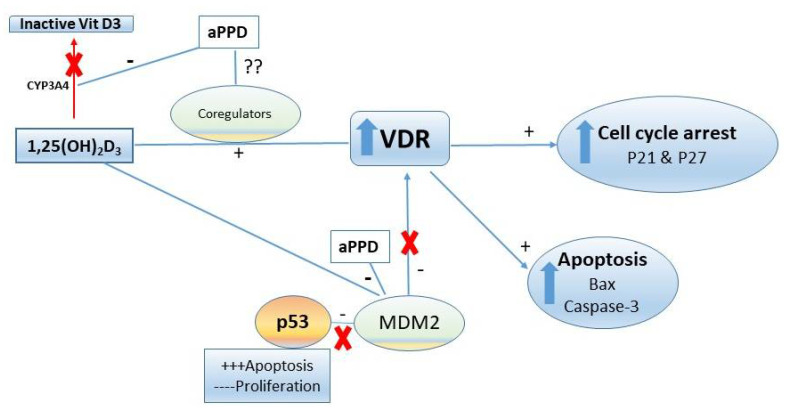
Proposed anti-prostate cancer activities mechanisms of aPPD in combination with 1,25-dihydroxyvitamin D3 (calcitriol). Due to the nature of their interactions, both pharmacokinetic (through CYP3A4) and pharmacodynamic (through VDR, MDM2) mechanisms could play a role in the enhanced antitumor activity of aPPD and calcitriol. VDR, vitamin D receptor; MDM2, mouse double minute-2; CYP3A4, cytochrome P450 3A4.

The VDR-mediated antitumor effects can be achieved through several downstream mechanisms including pro-apoptotic, cell cycle arrest, and anti-proliferative pathways (Figure 7). In prostate and other cancer types, several VDR-modulated genes can regulate the cancer development and progression. However, caspase-3 levels were similar in calcitriol-only group and combination group with opposing antitumor effects. It is critical to recognize that higher levels of caspase-3 do not guarantee antitumor effects or vice versa and, in contrast, depending on the tumor type and its complex microenvironment caspase-3 may play non-apoptotic roles in crucial tumorigenesis processes including cell proliferation, migration, or invasion [62]. Huang et al. (2011) reported that apoptotic tumor cells stimulate the repopulation of tumors from a small number of surviving cells [63]. Higher levels of caspase-3 in tumors were correlated with significantly stimulated tumor cell proliferation in vivo via compensatory proliferation for tissue regeneration mediated by prostaglandin E_3_ [63]. Similarly, Donato et al. (2014) reported that caspase-3 in dying melanoma cells significantly stimulated the growth of living melanoma cells in vitro and in vivo [64]. Calcitriol demonstrated antitumor effects in combination with CYP24A1 inhibitors in PC-3 prostate xenograft mouse in caspase-3-independent apoptosis pathways [49]. Additional reports suggest that caspase-3 is simply one of the many cell death or antiproliferative pathways that can facilitate antitumor effects of calcitriol in a tumor-dependent manner [65,66,67]. Indeed, we have previously shown in vitro that aPPD and calcitriol combination accomplishes the anticancer effects in androgen-dependent non-metastatic LNCaP and androgen-independent metastatic C4-2 cells through a variety of apoptotic and cell cycle pathways. The combination treatment increased the expression of pro-apoptotic Bax and amplifies the anti-apoptotic Bcl2 inhibition. Similarly, the combination was able to block the cdk2 cell cycle protein [21]. In certain cases, cells treated with aPPD alone demonstrated higher apoptotic and cell cycle protein expression than the calcitriol-alone group [21]. We postulate that the aPPD and calcitriol combination facilitates the antitumor action in the C4-2 human prostate cell xenograft model through non-caspase-3 mediated apoptosis (e.g., Bcl2, Bax) and cell cycle control (e.g., cyclin D1, cdk2, P21, and P27) pathways of VDR-mediated downstream effects. It is also plausible that both aPPD and calcitriol interacts through the mouse double minute-2 (MDM2) pathway [68,69]. MDM2 protein can strongly downregulate p53, which is a major tumor suppressor gene [70], and can also negatively affect VDR and its downstream functions [71]. Both aPPD and calcitriol can antagonize MDM2 protein, which can eventually lead to improved anticancer outcome through enhanced p53 and VDR functions (Figure 7).

Pharmacokinetic interactions between calcitriol and aPPD can lead to increased antitumor effects in vivo. We have shown that aPPD and calcitriol independently demonstrate anticancer effects in prostate cancer models [21,25]. Our lab has also shown that CYP3A4 is responsible for the hepatic metabolism and inactivation of calcitriol and aPPD is a CYP3A4 inhibitor [72,73]. The aPPD can inhibit the CYP3A4-mediated inactivation of calcitriol and thus increases bioavailability of calcitriol in vivo [42,72]. The single oral dose pharmacokinetic study with the concomitant administration of aPPD and calcitriol led to an elevated maximum serum concentration (Cmax), area under the curve (AUC), and delayed clearance in non-tumor bearing nude mice. Likewise, although there was a trend of increased calcitriol concentration starting with week one and then at week three, combinations of oral aPPD and calcitriol demonstrated significantly higher calcitriol serum concentrations compared to the calcitriol-alone group after six weeks of treatment in C4-2 xenograft mice [42]. In the present study, the antitumor efficacy was assessed for the same duration of treatment similar to our pharmacokinetic interaction research, which suggests that the enhanced antitumor effects of the combination in vivo may stem from elevated bioavailability of calcitriol (Figure 7).

## 5. Conclusions

The current work elucidated the antitumor effects of aPPD and calcitriol in combination on androgen-independent C4-2 xenograft CRPC mouse model in vivo. Treatment of tumor-bearing nude mice with aPPD and calcitriol significantly inhibited tumor growth with no signs of acute or chronic toxic effects. The mechanisms of antitumor effects were demonstrated through VDR protein overexpression, enhancement of VDR activation, and stimulation of apoptosis. Interestingly, the in silico docking study suggests that aPPD binds to non-active LBD site(s) but allosteric binding potentially increases VDR activity along with calcitriol. In conclusion, our results suggest the potential antitumor benefits of using calcitriol in combination with aPPD during castration-resistant prostate cancer, with limited or no toxicity.

## Data Availability

The data presented in this study are available on reasonable request from the corresponding authors.

## Supplementary Materials

The following are available online at https://www.mdpi.com/article/10.3390/medicines8060028/s1, Table S1: Measures of toxicity in androgen-independent C4-2 xenograft nude mice (*n* = 3).
