# Supplementary material for: Anti-Tumor Effects of Ginsenoside 20(S)-Protopanaxadiol and 1,25-Dihydroxyvitamin D3 Combination in Castration Resistant Prostate Cancer
Source: Medicines (Basel). 2021 Jun 4;8(6):28. doi: 10.3390/medicines8060028 (PMC8227560; doi:10.3390/medicines8060028)
Supplement: Supplementary file 1 [file medicines-08-00028-s001.zip › medicines-1182944-supplementary.pdf]

Supplementary Materials: Anti-tumor Effects of Ginsenoside 20(S)-Protopanaxadiol and 1,25-Dihydroxyvitamin D3 Combination in Castration Resistant Prostate Cancer

Mohamed Ben-Eltriki, Subrata Deb, Gehana Shankar, Gray Meckling, Mohamed Hassona, Takeshi Yamazaki, Ladan Fazli, Mei Yieng Chin and Emma S. Tomlinson Guns

Table S1. Measures of toxicity in androgen-independent C4-2 xenograft nude mice (n = 3). Mouse serum was used to carry out laboratory analyses. Data are presented as mean ± SEM. A p value < 0.05 was considered significant (*) change compared to control (vehicle-treated group). A p value < 0.05 was considered significant (* or #) and a p value < 0.01 was considered very significant (** or ##) change compared to vehicle control (*) or calcitriol- or aPPD-treated group (#).

Parameters/Units	Control	Calcitriol	aPPD	aPPD + Calcitriol
ALB (g/L)	44.5 ± 0.71	48.67 ± 1.15	47.33 ± 1.53	46 ± 1.01
ALP (U/L)	39 ± 11.31	36 ± 9.53	29 ± 7.55	27 ± 5.56
ALT (U/L)	48 ± 8.48	50.6 ± 14.74	43.5 ± 10.6	43.5 ± 3.29
AMY (U/L)	952 ± 54.02	1298.3 ± 114.6*	860.5 ± 14.85*	1332 ± 15.17**,##
LIP (U/L)	99 ± 36.01	92 ± 26.23	74.67 ± 14.52	82 ± 15.01
TBIL (μmol/L)	7 ± 0.05	6.33 ± 0.57	7.66 ± 3.01	6 ± 1.0
BUN (mmol/L)	5.25 ± 0.77	3.4 ± 0.49	4.73 ± 0.15	4.5 ± 0.78
CA (mmol/L)	2.565 ± 0.05	3.66 ± 0.24**	2.77 ± 0.06	3.3 ± 0.25*,#
PHOS (mmol/L)	2.28 ± 0.08	2.16 ± 0.42	2.37 ± 0.26	3.1 ± 0.63
CRE (μmol/L)	<18	<18	<18	<18
Glu (mmol/L)	8.7 ± 0.56	9.03 ± 0.83	9.5 ± 2.58	10.3 ± 3.29
NA (mmol/L)	154.5 ± 0.71	160 ± 1.02	158 ± 1.73	159.67 ± 3.21
K (mmol/L)	7.3 ± 0.28	7.36 ± 0.85	7.7 ± 0.85	7.65 ± 0.21
TP (g/L)	52.5 ± 0.71	58.67 ± 2.08	57.33 ± 0.57	56 ± 1.2
GLOB (g/L)	8 ± 0.65	9.67 ± 1.15	10.33 ± 2.08	10 ± 1.32

Abbreviations: Albumin (Alb), Alkaline Phosphatase (ALP), Alanine Aminotransferase (ALT), Amylase (AMY), Lipase (LIP), Blood Urea Nitrogen (BUN), Calcium (Ca), Phosphorus (PHOS), Creatinine (CRE), Glucose (Glu), Sodium (Na), Potassium (K), Total Protein (TP), Globulin (Glb). Gram (g), Litter (L), Mole (mol), Unit (U).
